# The reliability of the measurement of muscle volume using magnetic resonance imaging in typically developing infants by two raters

**DOI:** 10.1038/s41598-022-23087-y

**Published:** 2022-10-28

**Authors:** Georgia Whitta, Jessie Liang, N. Susan Stott, S. Ali Mirjalili, Malcolm Battin, Sîan A. Williams

**Affiliations:** 1 Department of Anatomy and Medical Imaging, Faculty of Medical and Health Sciences, University of Auckland, Auckland, New Zealand; 2 Department of Surgery, Faculty of Medical and Health Sciences, University of Auckland, Auckland, New Zealand; 3 Newborn Services, Auckland City Hospital, Park Road, Grafton, Auckland, New Zealand; 4 Curtin School of Allied Health, Faculty of Health Sciences, Curtin University, Perth, Australia; 5 Liggins Institute, University of Auckland, Private Bag 92019, Auckland, 1142 New Zealand

**Keywords:** Paediatric research, Musculoskeletal system

## Abstract

To assess intra-rater and inter-rater reliability of the manual segmentation of Magnetic Resonance Imaging (MRI) for the in vivo measurement of infant muscle volume of the knee extensor and flexor muscles by two raters. Muscles of the knee extensor and flexor muscle of ten typically developing infants (86 days ± 7 days) were scanned with MRI (Proton density sequence). Scans were then segmented using Slicer software, and volumes rendered by two raters. Intra-rater and inter-rater reliability were assessed using intra-class correlation (ICC), with mean difference (MD), standard error of the mean (SEM), and minimal detectable change (MDC) for each muscle calculated. ICCs for Intra-rater reliability of the segmentation process for the muscle volume of the muscles of the knee extensors and flexor muscles were 0.901–0.972, and 0.776–0.945 respectively, with inter-rater reliabilities between 0.914–0.954 and 0.848–0.978, for the knee extensor and flexors muscles respectively. For intra-rater reliability, MD ≤ − 0.47 cm^3^, MDCs for were < 1.09 cm^3^ and for inter-rater MD ≤ − 1.40 cm^3^, MDCs for were < 1.63 cm^3^ for all muscles. MRI segmentation for muscle volumes showed good to excellent reliability, though given the small volumes of the muscles themselves, variations between raters are amplified. Care should be taken in the reporting and interpretation of infant muscle volume.

## Introduction

Skeletal muscle function is determined by the intrinsic architecture and morphology of the muscle, such that muscles employed for fast and powerful bursts of force are structured differently (e.g. the soleus muscle) to muscles specialising in creating larger excursions over longer periods of time (e.g. the gluteus maximus)^1^. Variations in both muscle structure and functional outputs are also evident between different population cohorts, such as those of trained athletes^2^, and elderly cohorts^3^. Though differences within, and between, muscles may be evident at both the microscopic and macroscopic level, a gross measure such as muscle volume (which takes into account the full length and shape of the muscle) is suitably able to illustrate this link between muscle ‘make up’ and its function^4^.

Deficits in muscle volume are well documented in clinical populations experiencing impaired muscle function such as osteoarthritis, cerebral palsy, and post-musculoskeletal injury, with changes commonly linked with reduced outputs in muscle strength. For example, research on patients with osteoarthritis at the hip joint shows the involved muscles to be on average 22–26% weaker compared to age-matched peers, with the muscle volume reduced by 5–30%^5^. The link between muscle volume and strength has also been documented in children with and without cerebral palsy, with reductions in both strength and volume measured in children with cerebral palsy compared with typically developing peers^6^. Numerous studies have quantified the degree of loss of muscle bulk in this condition, with particular interest paid to the medial gastrocnemius muscle due to common presentations with plantarflexion contractures at the ankle^7^. Researchers have also sought to compare muscle volume and estimates of the rate growth between infants with cerebral palsy (mean age 35mo, range 8–65mo) and typically developing infants (mean age 29mo, range 1–69mo), finding variations in growth occurring as young as 15.5 months of age^8^. Further to this, studies also indicate the reductions in volume appear greater when the child’s gross motor function is more severely impacted^9,10^. Measures of muscle volume can thus be useful either as potential predictors of an individual’s muscular ability, or for the evaluation of interventions targeted at optimising muscle function (via changes in volume)^11–13^.

Magnetic Resonance Imaging (MRI) is generally considered the gold standard measurement of muscle volume in human muscle, due to the reliability and validity of this imaging modality^14,15^. The magnetic field within an MRI scanner creates a detailed image using the magnetic properties of different body tissues^16^, with post processing segmentation of the muscles to allow for the calculation of the muscle volume^17^. Manual segmentation involves tracing the outline of the muscle of interest from each MRI slice taken along the entire length of the muscle, and, though time consuming, has been shown to demonstrate good to excellent intra-rater reliability^14,18–20^ and moderate to good inter-rater reliability for muscle volume estimation^14,18–24^. However, much of the reliability, validation, and muscle volume quantification studies that have been conducted have focussed on older child (5–14 years old)^7^ or adult populations^15^. A scoping review by Williams et al. investigating the use of Ultrasound and MRI to analyse skeletal muscle architecture in individuals with cerebral palsy revealed that few existing studies included children under the age of 4 with cerebral palsy, with even less focus on the infant population^7^. The changes in growth and development during infancy commences the important trajectory for growth from childhood, adolescence, and into adulthood. Establishing a benchmark understanding for typically developing muscle growth in infancy is key to working towards early identification of impaired growth, that, if clinically indicated, may be circumvented by targeted intervention^8,25^.

Since MRI segmentation has not been used previously to calculate muscle volume of such a young population, it is crucial to establish the reliability of the manual segmentation of this measure, particularly given the small size of the muscles in this population and potential variation in anatomical parameters of the muscle during early stages of development. Authors of a 2018 systematic review commented on the reliability of quantifying muscle volume with MRI using manual segmentation techniques, noting that the variation of reliability between different individual muscles appeared to be based on segmentation challenges^15^. For example, it may be more difficult to identify the external borders of deeper muscles, and pathological muscles that have different shapes and indeterminate muscle boundaries^15^. Whilst automated segmentation techniques (partial or complete) have the potential to remove human error whilst also offering a more time efficient approach to segmentation, automation is not always an option for research teams, and can be limited in cohorts where an anatomical atlas based on large datasets are unavailable^17^. Establishing parameters of reliability for infant muscle growth will help determine the value of these measures in increasing our knowledge of infant muscle growth. Therefore, the aim of this study was to assess the intra and inter-rater reliability of the measurement of muscle volume of the knee extensor and flexor muscles using MRI in an infant population.

## Methods

### Study design

This reliability study investigated the manual segmentation of in vivo MRI scans of the lower limb in typically developing infants aged 0–6 months across two raters. The study procedures were approved by the University of Auckland Human Participants Ethics Committee (Reference number: 021155). This study was conducted in accordance with the Declaration of Helsinki.

### Participants

Recruitment of participants occurred between March 2019 and February 2020 through an email advertisement sent to staff of the University of Auckland Faculty of Medical and Health Sciences, through Facebook (Facebook (Meta Platforms, USA)) targeted advertisements, and through word of mouth. Families indicating their interest in the study were contacted by the research team and provided detailed information via email and a phone call. Full procedures and protocol of the study were reiterated face to face before written informed consent from the parent was obtained. Inclusion of infants required a normal Ultrasound scan at 20 weeks’ gestation and a term birth. Exclusion criteria included (i) infants born at < 36 weeks’ gestation; (ii) infants with a major congenital anomaly or health condition that affected foetal growth and was evident at birth; (iii) presence of a maternal health condition that impacted foetal growth.

### MRI set up and data collection

MRI scans were completed at the Centre for Advanced Magnetic Resonance Imaging (CAMRI) at the University of Auckland with an axial spin-echo Proton density sequence (3 T Siemens Skyra whole body MR unit). Interleaved multiscale 2D images were collected using a repetition time of 5400 ms, echo time of 38 ms, slice thickness of 3 mm, matrix size of 168 × 320 mm, and a field of view of 135–180 mm. Prior to the scan, participants were fed and swaddled with their legs in an extended position and were fitted with earplugs and baby muffs (with the positioning and fitting of the protective hearing apparel rechecked prior to the scan commencing). To assist the desired extended and neutral position of the legs in the scanner, foam tubing, padding, a vacuumed bean bag, and Velcro straps were utilised. However, the naturally preferred flexed position of infant legs meant that the legs were slightly externally rotated at the hip and flexed at the knee and hip joints despite efforts made by the team. The scan was conducted bilaterally from the level of the calcaneus to the iliac crest of participants as they lay supine in the scanner.

### Segmentation

**Figure 1:**
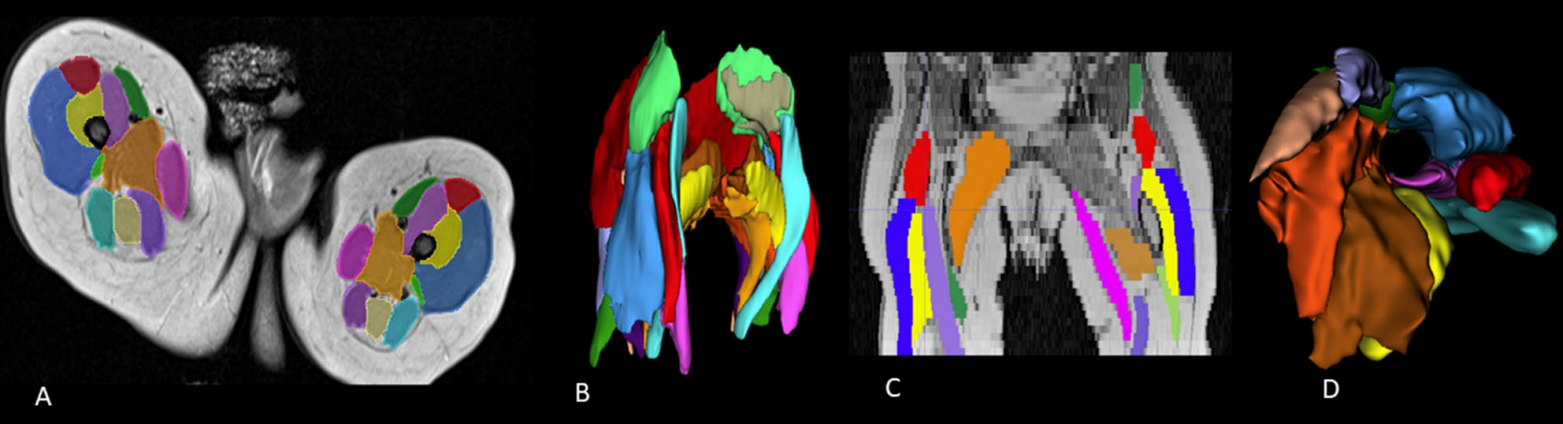
(**A**) Cross-sectional view of the muscles of an infant thigh, the outline of individual muscles are traced along its length to create a 3D model of the muscles. (**B**) 3D model from the anterior view. (**C**) Coronial view of the segmented muscles. (**D**) Superior view showing the path of the femur bone in the 3D model.

MRI scans of participants were transferred as DICOM files (Digital Imaging and Communications in Medicine) to open-source medical image processing software 3D Slicer^26^ to allow manual segmentation and calculation of muscle volumes. Two raters (GW and JL) first created a protocol ‘guideline’ (Supplementary 1) for the analysis and segmentation of each muscle in the Slicer software as part of their MRI segmentation training. This was aided by external resources^6,27^ that guided the raters in identifying muscle borders, local blood vessels and nervous structures, and the origins and insertions of muscle as seen on MRI scan. Raters were trained under the guidance of a supervisor experienced in Slicer segmentation of paediatric muscles. Some key considerations in the Slicer protocol included; (i) grouping together adductor magnus, adductor longus, and adductor brevis as one muscle mass due to the difficulty distinguishing muscle borders, a technique common with other studies^28^; (ii) excluding tendon attachments of muscles and only considering the muscle belly; (iii) considering the border of a muscle to be the outermost part of the muscle where the colour and contrast was distinctly different to that of the surround tissue. The 3D Slicer programme allows for visualisation and manual segmentation in axial, sagittal, and coronal views. After first marking muscle boundaries in the axial view, raters were then able to translate the MRI into sagittal and coronal planes to allow for correct muscle border selection and maximum voxel inclusion. Once muscle segmentation was completed, the ‘Segment Statistics’ module inbuilt into the 3D Slicer programme was utilised to calculate muscle volumes for each muscle that was manually segmented. DICOM files define the precise location and thickness of a MRI slice, allowing for the reconstruction of the muscles into a 3D model (Fig. 1). A combined total of over 800 h of manual segmentation was undertaken by both raters to complete the segmentation.

Muscles of the Knee Extensor muscle group (rectus femoris, vastus lateralis, vastus medialis), and the Knee Flexors muscle group (biceps femoris brevis, biceps femoris longus, semitendinosus, semimembranosus, and gracilis) were segmented for analysis. For intra-rater reliability analysis, a total of 10 limbs were segmented from 10 MRI scans (n = 9 participants for the knee extensor muscles (GW), n = 10 participants for the knee flexor muscles (JL), with segmentation repeated 2 months later. The discrepancy between the number of infants included in the extensor muscle and flexor muscle analysis was a result of independent reviewers’ scan selection. For assessment of inter-rater reliability, two raters independently segmented the muscles of interest from ten scans (n = 9 participants, one participant completing two scans 3 months apart), with limbs from five participants segmented bilaterally, and scans from five participants segmented unilaterally.

### Statistical analysis

All statistics were calculated with SPSS (IBM SPSS Statistics Version 26 for MacOS). The test–retest reliability of intra-rater and inter-rater measurements was analysed using intraclass correlation coefficients (ICC), with an ICC measuring below 0.5 rated as poor, between 0.5 and 0.75 moderate, 0.75–0.9 good, and greater than 0.9 rated as excellent reliability^28^. Box and whisker plots were used to provide a graphical representation of the inter-rater percentage variation in measures for different muscles, calculated as the difference in interrater measurement divided by the mean of the two measurements, with a Kruskal Wallis non-parametric ANOVA used to look for differences between group median percentages. Bland Altman analyses were used to further define the degree of bias in measures and calculate the 95% limits of agreement (LoA). Absolute reliability was defined using standard errors of measurement (SEM)’s which were calculated according to the equation

$${\text{SEM }} = {\text{ SD }} \times \, \surd \left( {{1} - {\text{r}}} \right){\text{ where r}} = {\text{ ICC}}$$

Minimal detectable change (MDC) was calculated using the formula (1.65 × √2 × SEM) for a 90% confidence interval^29^.

## Results

### Participants

Ten MRI scans for 10 infants (for intra-rater knee-flexor analysis) or 9 infants (for intra-rater knee-extensor analysis and inter-rater analysis) were included with a summary of infant characteristics outlined in Table 1.

**Table 1:** Participant demographics and anthropometric measures of participant subgroups.

	Knee extensor intra-rater	Knee flexor intra-rater
**Number of participants** (m:f)	n = 9 (5:4)	n = 10 (5:5)
**Age** (days) [range]	95.5 ± 28.4 [74–174]	86.5 ± 7.0 [74–174]
**Weight** (kg)	6.6 ± 1.3	6.3 ± 1.1
**Height** (cm)	62.0 ± 4.8	60.5 ± 3.2
**Muscle volumes** (ml)		
Rectus femoris	7.1 ± 1.8	
Vastus lateralis	22.5 ± 5.9	
Vastus medialis	11.8 ± 2.8	
Biceps femoris brevis		3.4 ± 1.1
Biceps femoris longus		5.0 ± 1.6
Semitendinosus		6.1 ± 1.8
Semimembranosus		5.9 ± 2.0
Gracilis		5.8 ± 1.6

Data are presented by mean ± standard deviation, calculated by the measurement of the second round of digitising for each rater.

### Intra-rater reliability and agreement

Intra-rater ICC ranged from 0.901 to 0.972 for the extensor muscles (excellent reliability), and 0.776–0.945 for the flexor muscles, indicating good to excellent reliability. For the extensor muscles, the MD between the first and second rating of measurements was ≤ − 0.4 cm^3^ and ≤ 4.8%, SEMs were ≤ − 0.45 cm^3^, and MDC ≤ 1.06 cm^3^. For the flexor muscles, the MD between the first and second rating of measurements was ≤ − 0.5 cm^3^ and ≤ 9.3%, SEMs were ≤ 0.47 cm^3^, and MDC ≤ 1.09 cm^3^. There was no statistically significant difference (*p* > 0.05) between ratings (Table 2).

**Table 2:** Intra-rater reliability measurements for the thigh muscles of infants aged 6 months and younger as measured by two raters; rater 1 (analysis of the knee extensor muscles) and rater 2 (analysis of the knee flexor muscles).

	Mean difference		Intra-rater reliability (ICC)	95% CI LoA (cm^3^)	SEM (cm^3^)	MDC_90%_ (cm^3^)
	(cm^3^)	(%)				
Rectus femoris	0.1 (± 0.5)	1.1	0.965	− 0.98 to 1.14	0.10	0.24
Vastus lateralis	0.2 (± 1.5)	0.9	0.972	− 2.82 to 3.23	0.26	0.60
Vastus medialis	− 0.4 (± 1.7)	− 3.7	0.929	− 2.54 to 1.67	0.45	1.06
Biceps femoris brevis	0.04 (± 0.7)	1.1	0.791	− 1.39 to 1.47	0.33	0.78
Biceps femoris longus	− 0.5 (± 1.0)	− 9.3	0.776	− 2.40 to 1.47	0.47	1.09
Semitendinosus	− 0.1 (± 1.0)	− 2.4	0.799	− 2.12 to 1.84	0.45	1.06
Semimembranosus	− 0.2 (± 0.7)	− 3.5	0.945	− 1.57 to 1.16	0.16	0.38
Gracilis	0.03 (± 0.8)	0.5	0.897	− 1.48 to 1.54	0.25	0.58

Percentage mean difference was calculated based on mean muscle volumes.

*LoA* limits of agreement, *SEM* standard error of measurement, *MDC* minimum detectable change.

### Inter-rater reliability and agreement

Inter-rater ICC ranged from 0.914 to 0.954 for the extensor muscles (excellent reliability) and 0.848–0.979 for the flexor muscles (good to excellent reliability). For the extensor muscles, MD between rater measurements was ≤ 1.4 cm^3^ and ≤ 7.6%, SEMs were ≤ 0.70 cm^3^, and MDC were ≤ 1.63 cm^3^. For the flexor muscles, MD between rater measurements was ≤ 0.4 cm^3^ and ≤ 8.7%, SEMs were ≤ 0.30 cm^3^, and MDC were ≤ 0.70 cm^3^. There was no statistically significant difference between raters’ muscle volume measurements (Table 3).

**Table 3:** Inter-rater reliability measurements for the thigh muscles of infants aged 6 months and under.

Muscle	Mean difference		Inter-rater reliability (ICC)	95% LOA (cm^3^)	SEM (cm^3^)	MDC_90%_ (cm^3^)
	(cm^3^)	(%)				
Rectus femoris	− 0.5 (± 0.9)	− 7.6	0.914	− 2.36 to 1.28	0.27	0.64
Vastus lateralis	1.4 (± 2.7)	6.2	0.933	− 3.90 to 6.70	0.70	1.63
Vastus medialis	0.5 (± 1.1)	3.9	0.954	− 1.73 to 2.65	0.24	0.56
Biceps femoris brevis	0.2 (± 0.7)	6.6	0.848	− 1.59 to 1.14	0.27	0.64
Biceps femoris longus	0.4 (± 0.7)	8.7	0.939	− 1.74 to 0.87	0.17	0.39
Semitendinosus	− 0.1 (± 0.9)	− 1.3	0.880	− 1.07 to 1.24	0.30	0.70
Semimembranosus	0.2 (± 0.6)	3.6	0.979	− 1.36 to 0.94	0.09	0.20
Gracilis	− 0.4 (± 0.9)	− 6.5	0.898	− 1.28 to 1.89	0.29	0.69

Percentage mean difference was calculated based on mean muscle volumes.

*LoA* limits of agreement, *SEM* standard error of measurement, *MDC* minimum detectable change. For mean difference, a −ve symbol indicates that rater 2, on average, reported a larger muscle volume than rater 1.

**Figure 2:**
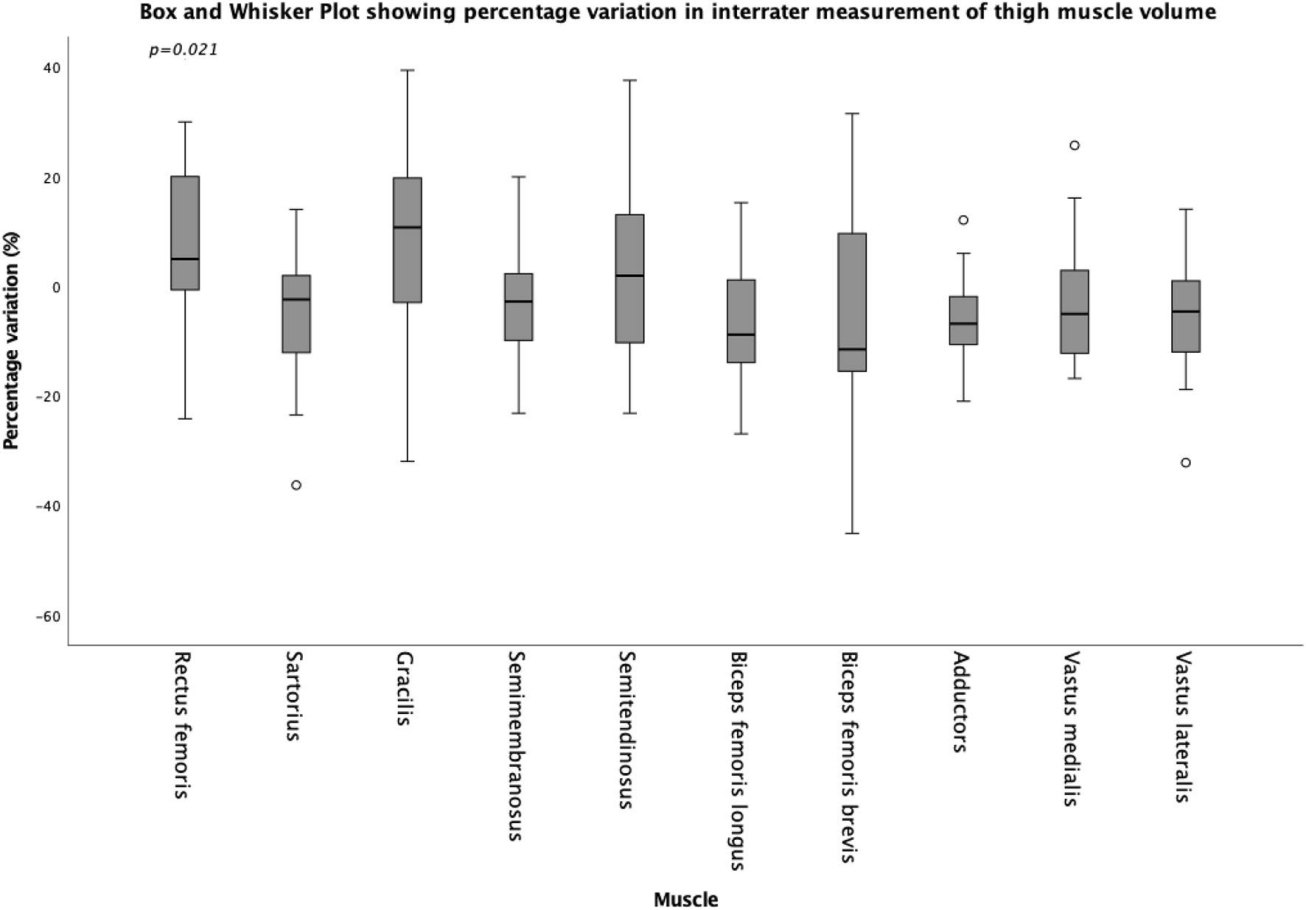
A box and whisker plot showing the percentage inter-observer variation for each individual muscle, with statistically significant difference (*p* < 0.05) between the group median percentages.

A box and whisker plot is presented in Fig. 2 to show the percentage inter-observer variation for each muscle, showing a statistically significant difference between the medians, with a Bland–Altman plot included within our supplementary material (Supplementary 2). Vastus lateralis showed the largest inter-rater LOA at − 3.90 cm^3^ to 6.70 cm^3^ with a bias of 1.40 cm^3^, and semitendinosus has the smallest inter-rater LOA of − 1.07 cm^3^ to 1.24 cm^3^ with a bias of − 0.08 cm^3^ (Table 3).

## Discussion

The aim of this study was to determine the intra and inter-rater reliability of manual MRI segmentation of the knee extensors and flexors muscles in an infant population. The relative intra-rater reliability was excellent for all knee extensor muscles and the semimembranosus (ICCs 0.901–0.972), and good for the remaining knee flexor muscles (ICCs 0.776–0.897). The mean differences were small for our two raters but, as a proportion of the muscle volume, ranged between 0.9–4.8%, and 0.5–9.3% for the extensor and flexors muscles respectively, with the highest variation in biceps femoris longus and vastus lateralis muscles. All muscles except the biceps femoris brevis, semitendinosus, and gracilis (ICCs 0.848–0.898) returned excellent inter-rater reliability (ICCs 0.914–0.979), however, the raw and percentage mean difference between raters indicate some challenges with segmenting infant muscle. Though broadly our data appear to support the use of segmented MRI data for the assessment of muscle volume in infant cohorts, caution is advised for muscles with difficult muscle boundaries that can be challenging to segment such as the Vasti muscles.

Previous studies analysing the reliability of manual MRI segmentation techniques in the analysis of lower limb muscle volumes have focused on older child and adult populations. Barnoiun et al. and Nordez et al. analysed the reliability of measuring the knee extensor muscles in adults using MRI scanning, with reporting excellent inter-rater reliability (ICC > 0.995 Barnoiun et al., ICC = 0.997 Nordez et al.) using slice-by-slice manual segmentation, and Nordez et al. also reporting excellent intra-rater reliability (ICC = 0.999)^18,30^. Noble et al. also reported excellent inter-assessor repeatability of the rectus femoris and semitendinosus (ICC 0.994 and 0.996), with mean differences of 4.7 ml (3.1%) and 4.5 ml (2.6%) respectively within adolescents and young adults with and without cerebral palsy (n = 7, 12–23 years)^31^. In agreement, the present study also demonstrated excellent intra and inter-rater reliability in the knee extensor muscles in our infant cohort, with small intra and inter-rater differences (intra: 0.1–0.4 cm^3^ and inter: 0.5–1.40 cm^3^). However, these differences should also be interpreted in the context of the size (i.e., volume) of the muscle itself with the intra-rater difference close to 4% for the vastus medialis muscle, and inter-rater differences ranging from 3.9 to 7.6%. These percentage differences appear to be slightly higher than that reported within older cohorts, but potentially still within an acceptable range. Handsfield et al., 2016 reported a mean intra-observer variability of 4.4%, and inter-observer variability of 4.7% across all muscles of the lower limb in children with and without cerebral palsy (11–17-year-olds)^32^. Similar to previous investigations on the reliability of muscle segmentation for volume, variations in reliability are apparent for specific muscles^30^, and in particular those muscles that may present challenges with the consistent identification of muscle borders, such as those with intimate borders like those seen within the knee extensor muscles. Given that the vastus lateralis and biceps femoris brevis share a border throughout the distal half of the femur, there is a possibility that portions of the biceps femoris brevis muscle may to be included in the segmentation of vastus lateralis or vice versa^30^. Discrepancies in the identification of the borders of Vasti muscles as also been discussed previously within the literature^30,33^. Willan et al. discussed in their findings from the segmentation of 40 adult cadavers that the vastus intermedius and vastus lateralis muscles sat on a continuum between completely fused to distinctly separated^33^. When vastus intermedius and vastus lateralis in an individual are partially fused, it makes segmentation difficult to determine. In addition to this, with such variation in the anatomy of these two muscles within the population, it is difficult to determine a standardised segmentation technique to apply to all muscles undergoing segmentation^33^.

**Figure 3:**
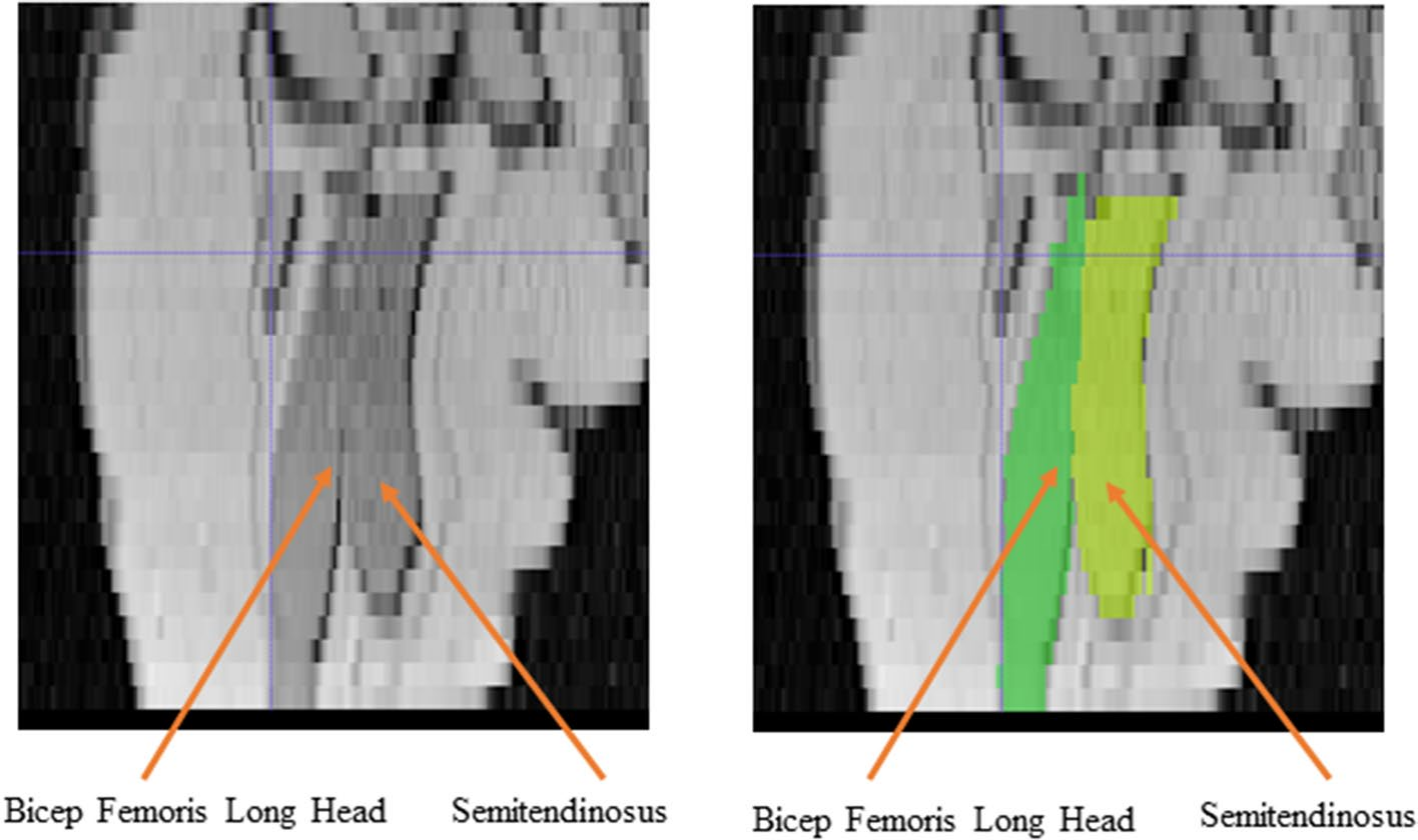
A coronal view of a left thigh (the posterior view) highlighting the challenges faced in segmenting muscles sharing intimate borders, here the proximal region of long head of the biceps femoris and the semitendinosus muscles.

Within the knee flexor compartment, biceps femoris longus had lower inter-rater ICCs, as well as higher levels of inter-rater and intra-rater bias. In addition, semitendinosus had decreased ICCs and inter-rater bias was higher. Again, these two muscles share intimate borders throughout the length of the thigh, and also share a common origin on the inferomedial ischial tuberosity, proving difficult to distinguish the muscle bellies from one another^34^ (Fig. 3). In general, segmentation in the muscles of the knee flexor group appeared more varied, though all reported to have good to excellent reliability. Comparatively, the reliability in older children (aged 5–11 years old) appears to be stronger, with Pitcher et al. and Williams et al. reporting excellent intra-rater (ICC > 0.99 in Pitcher et al. and ICC = 0.97 in Williams et al.) and inter-rater (ICC > 0.99 for Pitcher et al.) reliability for the muscle volume measurement of the knee flexor muscles^9,35^. Though it should also be noted that in the current study, participants lay supine (not prone like the older children), potentially applying added compression to the flexor muscles and adding an additional challenge to border determination.

Similar to findings in previous literature in older cohorts^18,32,36^, we observed a common pattern of larger variations/bias in inter-rater reliability compared with intra-rater testing. This is indicative of the influence and importance of interpretation and identification of muscle borders. It has been suggested previously that systematic differences in the determination of muscle-fascia borders and muscle origins and insertions are likely to result in greater inter-rater variability^15^, as such, establishing robust segmentation guidelines prior to scanning may aid in the reliability of segmentation to increased confidence in the comparison of data between different research groups. Analysing muscle volumes as an anatomical functional group (in addition to presenting individual muscle data) may also reduce the variability seen when undertaking manual segmentation, particularly of muscles that share intimate borders^37^.

As part of this study, we calculated the 90% minimum detectable change (MDC), which indicates change outside of variations that are expected by chance within muscle volume measures. For all the extensor muscles, the MDC was < 10% of the mean measure of muscle volume, but was larger (up to > 20%) for the smaller flexor muscles. It also is important to note that an MDC is not always the same as a minimum clinically important difference, and further investigations are needed to aid our understanding of how much change would be clinically significant. This significance likely varies across muscles, ages, and the presence of neurologic disorders, but is important in the interpretation of any documented change.

## Limitations

Infant MRI presents additional challenges for obtaining successful (i.e., readable) scans. Even whilst sleeping and with strapping and foam padding, an infant can still move within the scanner, and may attempt to bend up the legs to move into a preferred flexed (i.e., curled up) posture. Allowing ample time to reposition the infant in the MRI, add foam padding and Velcro strapping, and time to repeat the MRI sequences is recommended. Our sample size was determined by the availability of successful infant MRI scans within the time frame of the study, as such one infant scan was not used due to poor quality and indeterminate borders. This study only focused on muscles of the thigh as identified as a particular gap within the available literature, with most studies appearing to focus on muscles of the lower leg^8,38^. The focus of this study was on the manual segmentation of infant muscle, which we approached using two raters. Partial or complete automatic segmentation processes with additional raters could be investigated in future studies to allow for stronger statistical inference of this work to be drawn.

## Conclusion

Though the data presented indicate MRI segmentation to be a reliable and acceptable approach to the measurement of thigh muscle volume in infants under 6 months of age, caution is still advised when capturing MRI data in this age group. The quality of infant scans can provide significant barriers to scan interpretation, therefore methods to improve this issue are a priority for working in this area. Measuring infant muscle size in vivo can enable clinicians to monitor infantile muscle growth, with the potential to identify variations or impairments in muscle growth, as well as evaluating the effects of potential therapeutic interventions.

## Supplementary Information

Supplementary Information 1.

Supplementary Information 2.

Supplementary Legends.

## Data Availability

The datasets used and/or analysed during the current study available from the corresponding author on reasonable request.
