# Supplementary material for: The reliability of the measurement of muscle volume using magnetic resonance imaging in typically developing infants by two raters
Source: Sci Rep. 2022 Oct 28;12:18191. doi: 10.1038/s41598-022-23087-y (PMC9616850; doi:10.1038/s41598-022-23087-y)
Supplement: Supplementary file 1 — Supplementary Information 1. [file 41598_2022_23087_MOESM1_ESM.pdf]

GUIDANCE FOR THE MANUAL DIGITISING OF INFANT THIGH MUSCLES

A guide of the decisions made by two independent analysers when segmentation boundaries of muscles on MRI scans using Slicer software.

The guidance provided by '*Handsfield et al. (2014) "Relationships of 35 lower limb muscles to height and body mass quantified using MRI." J Biomech 47(3): 631-638.*' was a valuable reference guide for initial identification of muscle boundaries, guiding the team initially in distinguishing and identifying the different lower limb muscle's positioning on an MRI scan. However, it was quickly observed that the anatomy of the infant muscles differed from the mostly adult MRI scans available within previous anatomy atlas' and that presented by Handsfield et al. As such, our team developed a set of rules and guidelines to aid in identifying, distinguishing and manual segmented each of these infant muscles from MRI scans.

Enclosed in this manual are general guidelines and rules developed by the research team, and more specific guidance for digitising the muscles of the Knee Extensor and Flexor muscles groups.

- Rectus Femoris
- Vastus lateralis, intermedius, and medialis
- Sartorius
- Gracilis
- Adductor magnus, longus, and brevis
- Semimembranosus
- Semitendinosus
- Biceps long head
- Biceps Short Head

GENERAL GUIDELINES

- Efforts made to exclude tendon attachments of muscles in the muscle volume. Care is to be taken at the proximal and distal borders of the muscle to differentiate between muscle belly and tendon. Assistance by using the contrast function on Slicer software to visualise the difference between these two tissue types.
- The quality of scans and pixel size sometimes meant that we could not accurately divide borders between adjacent muscles. In this case, the smaller muscle would be granted the muscle border in its volume estimate.
- Difficulty in distinguishing two muscles and their fascia border were common, in particular for muscles of the Vasti group and adductors. There was much discrepancy between different online sources and the Handsfield atlas regarding location of muscle attachment to bone and borders. Therefore, to ensure maximum interobserver and intra-observer accuracy, the decision was made to group Vastus lateralis, intermedius, and medialis as one muscle mass, and also group adductor magnus, longus, and brevis as one mass.
- Muscles were digitised from the most distal border of the most distal muscle to the most proximal border of the most proximal muscle.
- Since Slicer permits boundaries to be drawn by pixels, it was often necessary to overdraw the edges of the muscle slightly so that the border was fully encompassed (i.e. when the pixel sizes were too large). To ensure a consistent approach, the border was considered the outermost part of the muscle where the colour and contrast was distinctly different from the surrounding tissue.
- All axial image edges bloomed to the palest grey pixel.

MRI Segmentation process using Slicer

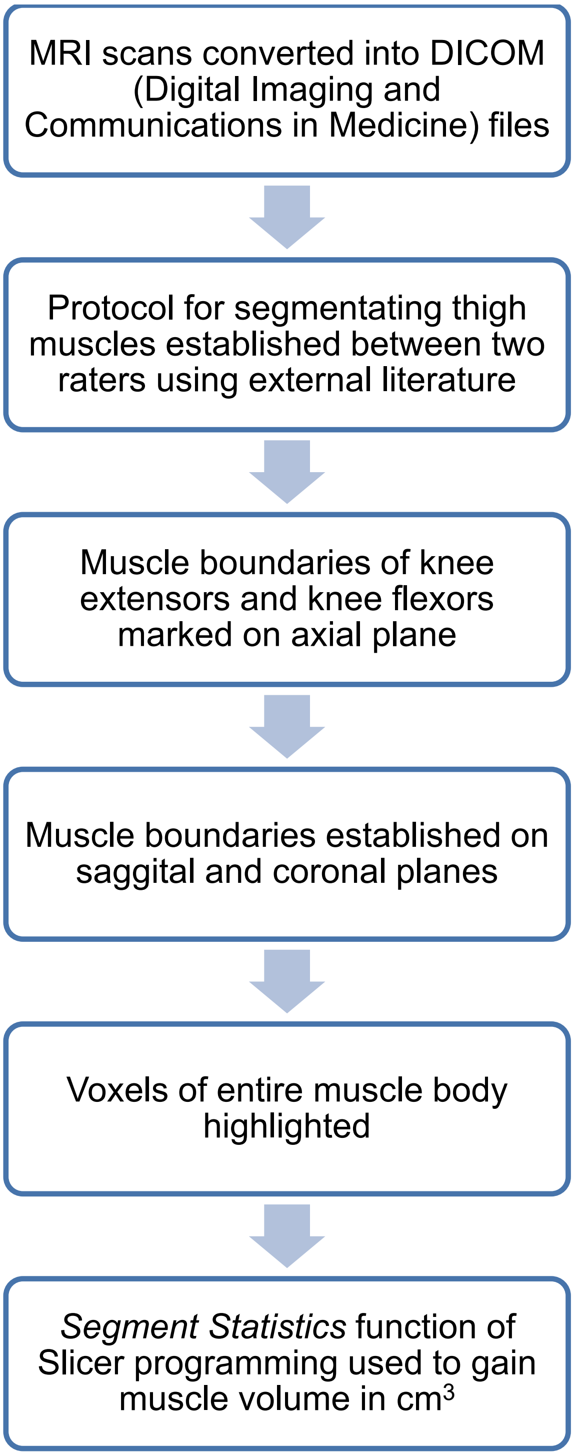

Rectus femoris

- Trace inferiorly, until quads tendon is apparent- using sagittal view to confirm this. Superiorly, until it disappears into gluteus minimus.

Here, the Rectus Femoris muscle is seen in red. This is illustrating how the sagittal view allows us to accurately visualise the superior and inferior reach of the Rectus Femoris muscle.

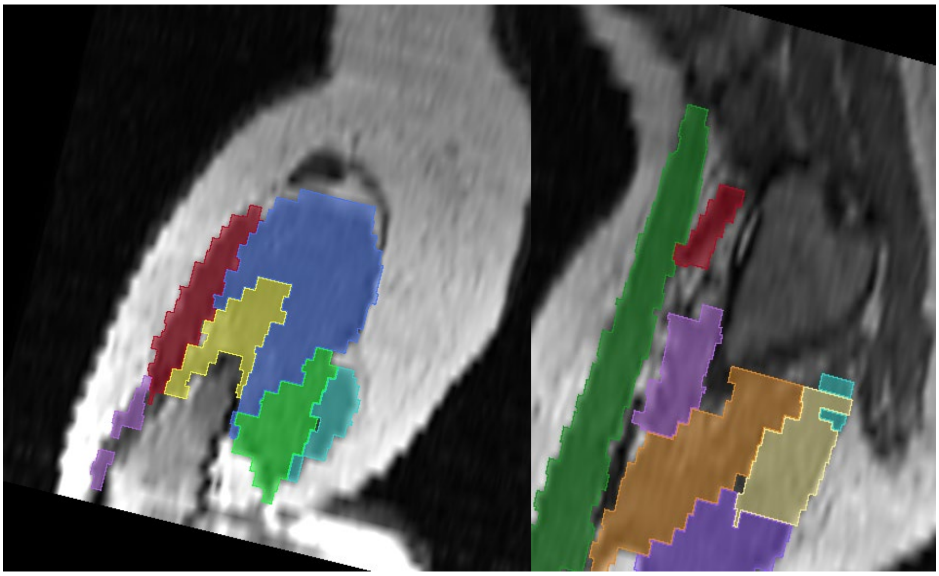

Vastus lateralis, intermedius, and medialis

- Iliotibial band excluded upon at least one voxel's space between it and vastus lateralis along the entire fascia.
- Biceps Femoris Brevis and Vastus Lateralis borders have been noted to be variable between raters.
- Border with femur is given leniency in favour of muscle.

These images are to show how the border of the femur and muscle interface is considered. The darker colour of the femur is 'cut into' to allow the muscle volumes to be fully considered.

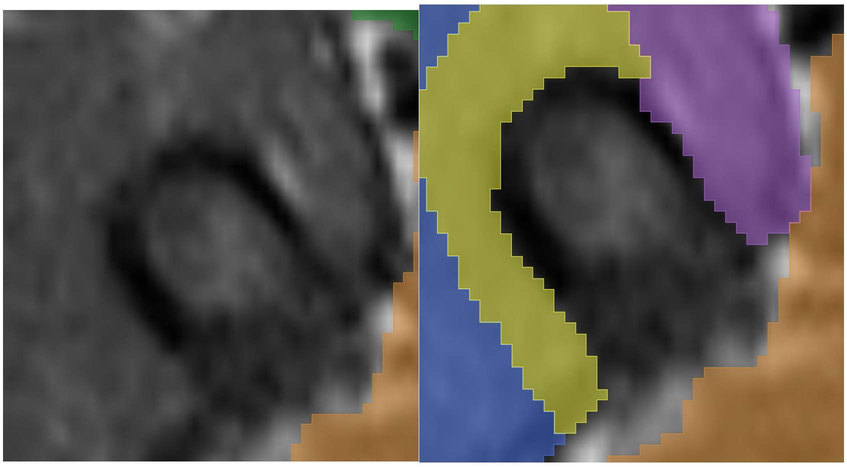

- Compartmental fascia between vasti and adductors is included in vasti volume due to inconsistent clarity across scans.
- Care is taken to acknowledge the presence of the femoral canal, white space surrounding the vessels is not encroached by segmentations and the vessels themselves distinguished from the border of the adductors.

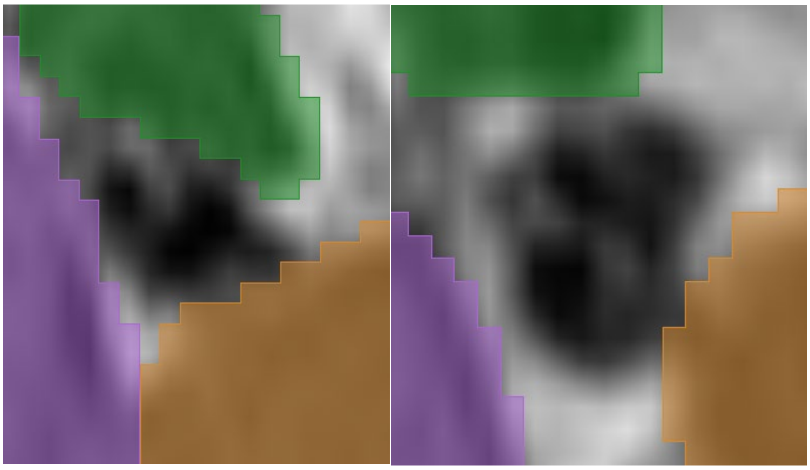

- Superiorly, note that gluteus medius which is similarly shaped comes in laterally at the very superior aspect of vastus lateralis. Other muscles disappear into femur.
- Inferiorly, the suprapatellar bursa and surrounding fat is acknowledged to take up the white space that appears to separate the femur and the vasti.

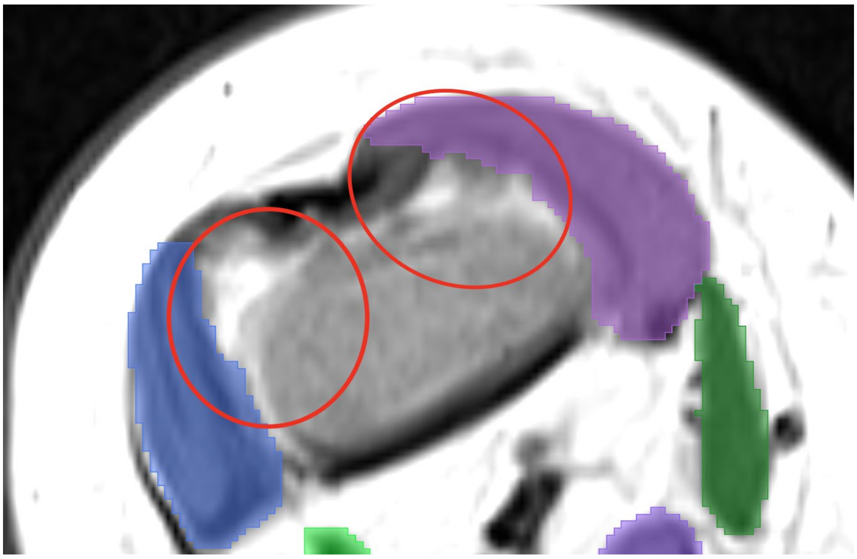

- It is noted that the inferior insertions of many muscles, including the vasti, are cut off and thus there is an unpredictable underestimation of volume.

Sartorius

- Inferiorly, the tendon is followed further than necessary due to the early cut-off.
- Superiorly, once the Anterior superior iliac spine/iliacus comes into view the most superior aspect of the sartorius is assumed.

Gracilis

- Superiorly, once it is indistinguishable it's volume is assumed to be part of the adductors.
- Inferiorly, the tendon is followed further than necessary due to the early cut-off.

Adductor magnus, longus, and brevis

- As previously stated, we grouped these three muscles together as one muscle mass due to the difficulty we had locating specific muscle borders.
- Superiorly, the origins disappears anterior-medially. Approaching the origins, care is taken to acknowledge encroachment by obturator externus posteriorly and pectineus medially.
- Care to exclude the sciatic nerve/vessel bundle posteriorly is taken.
- Anteriorly the femoral canal is also to be excluded.
- Due to amorphous nature the borders of the adductors are noted to be especially variable.

Semimembranosus

- Inferiorly, the tendon is followed further than necessary due to the early cut-off.
- Superiorly, it is followed until indistinguishable from surrounding structures, which is noted to be difficult due to the abundance of new structures appearing at this level.

Semitendinosus

- Inferiorly, the tendon is followed further than necessary due to the early cut-off.
- Superiorly, it is followed until indistinguishable from surrounding structures, which is noted to be difficult due to the abundance of new structures appearing at this level.

Biceps long head

- Inferiorly, the tendon is followed further than necessary due to the early cut-off
- Superiorly, it is followed until indistinguishable from surrounding structures, which is noted to be difficult due to the abundance of new structures appearing at this level.

Biceps short head

- Superiorly, the only indication of a border between Biceps Femoris Brevis and Vastus Lateralis is variation in gradient and therefore this is noted to be a site of variability between raters.
- Inferiorly, the tendon is followed further than necessary due to the early cut-off.
