# Supplementary figures and images for: The reliability of the measurement of muscle volume using magnetic resonance imaging in typically developing infants by two raters
Source: Sci Rep. 2022 Oct 28;12:18191. doi: 10.1038/s41598-022-23087-y (PMC9616850; doi:10.1038/s41598-022-23087-y)

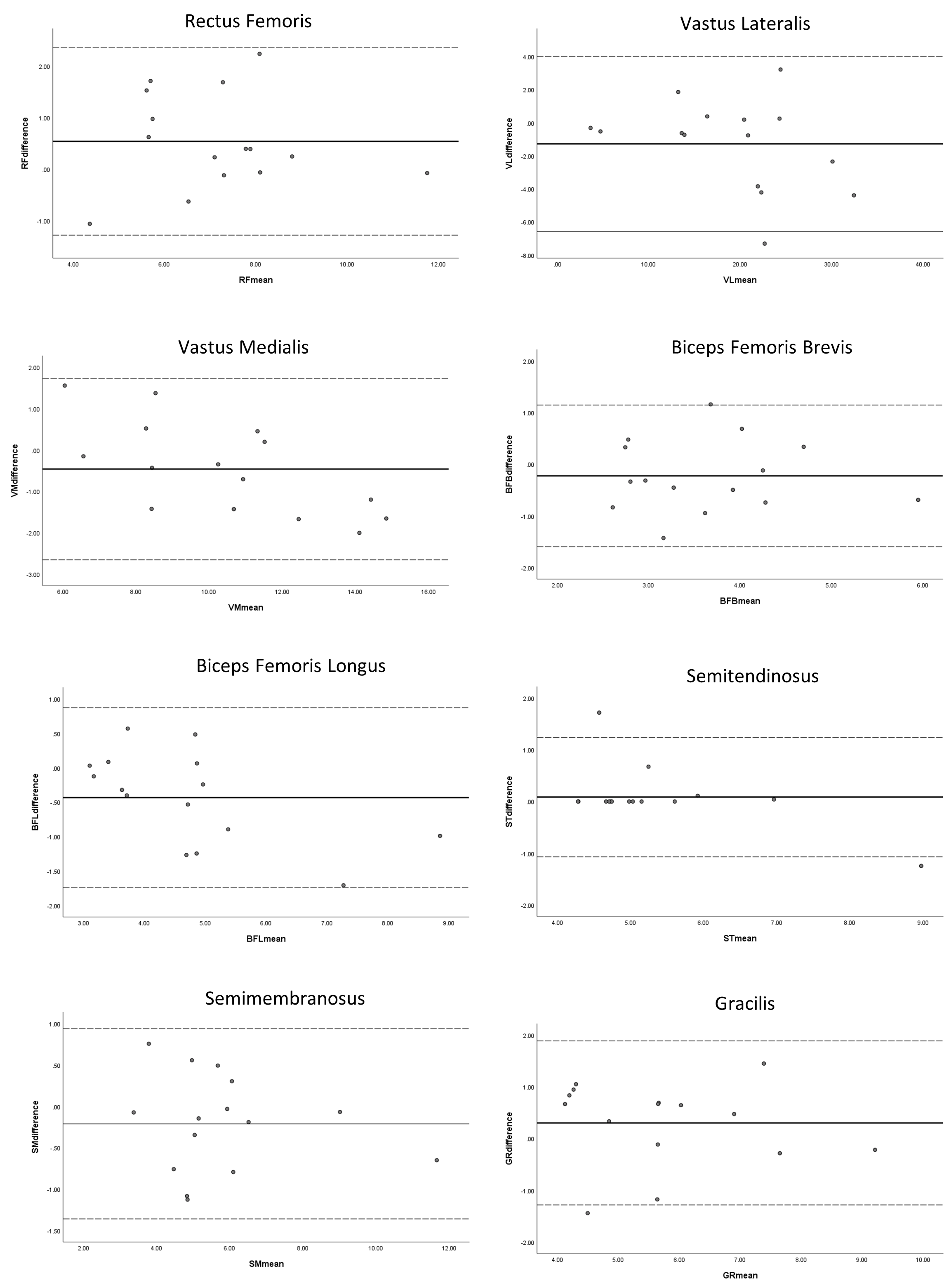

Supplement: Supplementary file 2 — Supplementary Information 2. [file 41598_2022_23087_MOESM2_ESM.png]
