# Supplementary material for: The reliability of the measurement of muscle volume using magnetic resonance imaging in typically developing infants by two raters
Source: Sci Rep. 2022 Oct 28;12:18191. doi: 10.1038/s41598-022-23087-y (PMC9616850; doi:10.1038/s41598-022-23087-y)
Supplement: Supplementary file 3 — Supplementary Legends. [file 41598_2022_23087_MOESM3_ESM.doxx]

**Supplementary 1:** Guidance for the manual digitising of infant thigh muscles.

**Supplementary 2:** Bland-Altmann Plots demonstrating inter-rater LOA and bias
